# Tracking Functional Brain Changes in Patients with Depression under Psychodynamic Psychotherapy Using Individualized Stimuli

**DOI:** 10.1371/journal.pone.0109037

**Published:** 2014-10-02

**Authors:** Daniel Wiswede, Svenja Taubner, Anna Buchheim, Thomas F. Münte, Michael Stasch, Manfred Cierpka, Horst Kächele, Gerhard Roth, Peter Erhard, Henrik Kessler

**Affiliations:** 1 Department of Neurology, University of Lübeck, Lübeck, Germany; 2 Department of Psychology, Alpen-Adria-University of Klagenfurt, Klagenfurt, Austria; 3 Institute of Psychology, University of Innsbruck, Innsbruck, Austria; 4 Institute of Psychosomatic Cooperation Research and Family Therapy, University of Heidelberg, Heidelberg, Germany; 5 Department of Psychosomatic Medicine and Psychotherapy, University of Ulm, Ulm, Germany; 6 Brain Research Institute, University of Bremen, Bremen, Germany; 7 Department of Psychosomatic Medicine and Psychotherapy, LWL University Hospital, Ruhr-University Bochum, Bochum, Germany; 8 Hanse Institute for Advanced Study, Delmenhorst, Germany; University of Electronic Science and Technology of China, China

## Abstract

**Objective:**

Neurobiological models of depression posit limbic hyperactivity that should normalize after successful treatment. For psychotherapy, though, brain changes in patients with depression show substantial variability. Two critical issues in relevant studies concern the use of unspecific stimulation experiments and relatively short treatment protocols. Therefore changes in brain reactions to individualized stimuli were studied in patients with depression after eight months of psychodynamic psychotherapy.

**Methods:**

18 unmedicated patients with recurrent major depressive disorder were confronted with individualized and clinically derived content in a functional MRI experiment before (T1) and after eight months (T2) of psychodynamic therapy. A control group of 17 healthy subjects was also tested twice without intervention. The experimental stimuli were sentences describing each participant's dysfunctional interpersonal relationship patterns derived from clinical interviews based on Operationalized Psychodynamic Diagnostics (OPD).

**Results:**

At T1 patients showed enhanced activation compared to controls in several limbic and subcortical regions, including amygdala and basal ganglia, when confronted with OPD sentences. At T2 the differences in brain activity between patients and controls were no longer apparent. Concurrently, patients had improved significantly in depression scores.

**Conclusions:**

Using ecologically valid stimuli, this study supports the model of limbic hyperactivity in depression that normalizes after treatment. Without a control group of untreated patients measured twice, though, changes in patients' brain activity could also be attributed to other factors than psychodynamic therapy.

## Introduction

In the search for neurobiological correlates of depression we have an increasingly clear picture of which brain areas typically show hyperactivity (e.g., amygdala, striatum and other limbic and subcortical regions) [1], [2], [3]. More recently, depression has been conceptualized in terms of network dysfunctions including cortico-limbic loops [3], [4], [5]. Consequently, those patterns of hyperactivity should normalize after treatment concurrently with clinical improvement [5], [6]. For psychotherapy, though, the neural correlates of treatment effects in depression are less well understood [7], [8], [9], [10], [11]. Regarding changes in limbic and subcortical regions, for instance, increases as well as decreases have been reported after psychotherapy [8], [11].

Two critical issues may contribute to this inconsistent picture [11], [12]. First, many studies used unspecific resting state measurements before and after treatment or standardized emotional stimuli rather than specific symptom provocation. Second, psychotherapeutic treatments may have been too short (typically 6–16 weeks) to lead to psychological changes instantiated in either brain activity or structure. To address the first issue, we conducted a previously published initial study in patients with depression using individualized symptom-specific stimuli as described below [13]. The second point, assessing functional brain changes during long-term psychotherapy, is covered by the present communication.

To enhance the ecological validity of stimuli and thus increase the chance to detect treatment effects, we created sentences describing each patient's current dysfunctional interpersonal relations [13] derived from clinical interviews based on Operationalized Psychodynamic Diagnostics [14]. For control participants, sentences described major sources of personal distress without being attached to depression. These stimuli, compared to emotionally negative control sentences, led to activation in medial prefrontal cortex, anterior cingulate cortex and bilateral occipital cortex in patients and healthy controls. Compared to controls, patients showed enhanced activation in areas confirming the notion of limbic hyperactivity in depression [1], [2], [3] (amygdala, caudate nucleus and putamen) and other regions (parts of the inferior, medial and precentral/middle frontal gyrus, postcentral gyrus) [13].

Extending this earlier work, we conducted a longitudinal study with the subjects included in the previous publication [13] with repetition of the same experiment after eight months of psychodynamic therapy. For comparison, the group of healthy individuals from our previous study was also assessed with parallel tasks after eight months. The long observation period of eight months, the exclusive application of psychotherapy (no concurrent medication) and the application of an established diagnostic tool from depression therapy for stimulus generation constitute an advance over previous studies.

In addition to clinical improvement evidenced by a reduced score on the Beck Depression Inventory (BDI) we expected the treatment to lead to a reduction of the relative limbic hyperactivity found prior to therapy [13]. For the control group we neither expected a change in depression scores nor in brain activity.

## Methods and Materials

### Participants

Participants were the same as in our previous study [13] and comprised 18 unmedicated patients (age 39.8 years SD 12.8, 14 women) with recurrent major depressive disorder and 17 healthy controls (age 38.0, SD 11.6, 14 women). Patients were recruited from a psychoanalytic institute and diagnosed by two trained clinicians using the Structured Clinical Interviews I and II for DSM-IV (German version, [15]). They reported between 1 to 15 depressive episodes (M[SD]  = 5.6[5.5]), and their age at first occurrence of depression was between 8 and 40 years (M[SD]  = 19.3[8.2]). Some patients had received various types of medication and psychotherapies during the course of their disease but had not received treatment within at least 6 months prior to inclusion in the study. Hence, the sample was completely free of psychotropic medication at the time of study. Exclusion criteria were other psychiatric conditions, substance abuse, significant medical or neurological conditions or eye problems. Control participants were matched for age, sex and education, and had no history of previous depressive episodes or other psychiatric conditions, including bipolar disorder (SCID). All participants were right-handed. In both groups, depression severity was assessed using the Beck Depression Inventory [16]. All participants were examined twice (mean time between first and second measurement in weeks: patients 33, range 29–34, controls 32.7, range 29–37). During this period, controls received no intervention while patients underwent psychotherapy by fully-trained, state-licensed and experienced therapists (n = 15) with a mean of 22.4 years (SD = 7.9) of experience in practicing psychotherapy in their respective private practice. A psychodynamic approach with a focus on intrapsychic conflicts and dysfunctional interpersonal relations represented in the OPD sentences used for stimulation (see below) was employed consistently for all patients. To foster adherence, the study therapists met monthly for group intervisions on a regular basis. Group intervision sessions were audiotaped for adherence control and assessing how therapies were affected by research [17]. All therapies were paid by the German health care system. The measuring time points will be referred to as T1 (first fMRI session, before therapeutic intervention for patients) and T2 (second fMRI session, after eight months of therapy for patients and eight months of waiting for controls). All participants gave written informed consent after complete description of the study and prior to their inclusion.

The study protocol had been approved by the ethical committee of the University of Ulm and was in compliance with national legislation, the principles expressed in the Declaration of Helsinki, and the Code of Ethical Principles for Medical Research Involving Human Subjects of the World Medical Association.

### fMRI Stimuli

Individualized stimuli were generated based on an interview according to the system of Operationalized Psychodynamic Diagnostics (OPD) [14] conducted by a trained clinician (HeK). Videotaped material was rated independently by 2–3 expert raters (OPD-Trainers). Typical dysfunctional interpersonal relations were identified and served as basis for the stimuli (“OPD sentences”). For patients sentences described a current problematic interpersonal relation typical of their depressive cognitions. For controls, sentences represented a major source of interpersonal distress without being connected to clinical depression. Four sentences were selected representing the typical dysfunctional relationship theme of each person (e.g., “You wish to be accepted by others.”, “Therefore you do a lot for them.”, “That is often too close for them, so they retreat.”, “Then you feel empty and lonesome.”). These individual sentences served as stimuli during both fMRI-sessions (OPD condition). Word count and semantic structure of the stimulus sentences did not differ between patients and controls (average word count of the four sentences, Controls: 31 words, Patients: 33 words, T(33)  = 1.1; n.s.). The control condition (“traffic”) comprised four sentences, which described a stressful traffic situation (“The other driver makes a mistake.”, “You are very upset about this.”, “You react to the other driver.”, “But he reacts inadequately.”). Prior to testing, participants were asked to remember a recent and stressful situation they had experienced in traffic. The rationale behind this control condition was to induce negative emotions and recall autobiographical memories with a personally relevant situation including human interactions, but without engaging in specific depression-related, clinically relevant material. In order to separate the two conditions (OPD and traffic), and let subjects calm down after emotionally demanding sentences, “relaxation” sentences were inserted between conditions. Those sentences instructed participants to relax. Whereas the OPD sentences were derived individually for each person, “relaxation” and “traffic” were the same across all subjects and repetitions. OPD sentences were slightly but significantly longer (M[SD]  = 49.8 [9.1] characters) than “traffic” sentences (43.5 characters). The stimuli were exactly the same for T1 and T2.

### fMRI Experiment

Sentences were presented by a projector onto a screen watched by the participants via a mirror while lying in the scanner. The four sentences of a condition (OPD, traffic, relaxation) were presented for 7.5 seconds each, resulting in 30 seconds blocks. During the OPD block participants were asked to mentally engage in situations with significant others, as described by the OPD sentences. They received no instruction to regulate their emotions, but should let spontaneous thoughts, emotions and memories come to mind. “Traffic” and “relaxation” conditions also comprised four sentences with each lasting 7.5 seconds. The instructions were to mentally engage either in the traffic situation or to relax. In total 12 “relaxation”, 6 “traffic” and 6 “OPD” blocks were presented (white Arial font, size 16, black background). Blocks were separated by a 5-seconds fixation cross. The entire experiment lasted approximately 15 minutes.

### Study Procedure

Four to six weeks prior to T1 fMRI assessment, all participants (patients and controls) were interviewed (SCID I+II, OPD) and filled out questionnaires (BDI) and informed consent forms. At the beginning of the fMRI session, they were briefed, saw their individual OPD sentences prior to actual scanning and were asked, whether the sentences fit and enticed them to think about their problematic relations. After scanning participants assessed the extent to which the OPD sentences were adequately describing their problematic relations and caused emotional arousal on a 7-point Likert scale. The scanning procedure and questionnaires were the same at T2.

### Image Acquisition

Data were obtained using a 3T SIEMENS Magnetom Allegra head scanner (Siemens, Erlangen, Germany). Participants were positioned on the scanner couch and wore foam earplugs to reduce scanner noise. An experienced psychotherapist not involved in the therapy of the patients (ST or HeK) assisted with the setup procedure and coached the participants throughout the experiment. Data acquisition started with anatomical images (3D high resolution T1-weighted isotropic volume, MPRAGE-sequence (MPRAGE  =  Magnetization Prepared Rapid Gradient Echo); TR = 2.3 s, FOV = 256×256×176 mm^3^, TE = 4.38 ms, TI = 900 ms, flip angle  = 8°, 1 mm isovoxel, total acquisition time 14.45 min). Functional scans were performed using a single shot echo planar imaging sequence (EPI). A total of 365 T2*-weighted whole brain volumes were acquired (EPI-sequence; TR 2500 ms, TE 30 ms, flip angle 90°, FOV = 192 mm, matrix 64×64, 44 slices, slice thickness 3 mm, interleaved acquisition order, AC PC- Orientation, total acquisition time: 15.18 min).

### Image Analysis

Data were analyzed and visualized using Brain Voyager QX 1.10 to 2.2 (Brain Innovation, Maastricht, Netherlands). Preprocessing: Functional data were slice-time corrected and motion was corrected relative to the first volume of the run. To remove low frequency drifts, data were high-pass filtered (3 cycles, three sine waves fall within the extent of the data). Structural and functional data were transformed into the standard space of Talairach and Tournoux [18], data points were labeled using Talairach Daemon [19]. The design matrix was modeled using the two gamma hemodynamic response function. Functional data were smoothed using an 8 mm full width at half maximum (FWHM) isotropic Gaussian Kernel. Group data were analyzed using random effects analyses based on z-transformed functional data. An ANOVA, including the within-factors CONDITION (OPD vs. traffic sentences), TIMEPOINT (T1 and T2) and between-factor GROUP (patient vs. control) was performed to identify differences in hemodynamic response. Motion-correction parameters were included in the GLM-Model as regressors of no interest.

Since we were interested in whether brain responses to individualized stimuli changed over time, we conducted a region-of-interest (ROI) analysis based on all clusters found to be significant in the GROUP × CONDITION interaction at T1 [13]. Those regions included parts of the right inferior frontal gyrus, the right amygdala, the medial frontal gyrus, the bilateral putamen, the precentral/middle frontal gyrus and the postcentral gyrus. In the ROI analyses, we tested the CONDITION × TIMEPOINT × GROUP interaction. To keep comparability with previous measurements at T1 [13], statistics were conducted and maps are shown with a threshold of p<0.001, uncorrected. A cluster size threshold of 16 voxels was consistently applied. Due to a lower risk of type I error, the ROI- analysis is reported with a threshold of p<.05 with correction for multiple comparisons based on False Discovery Rate (FDR) [20]. All active voxels are displayed in native resolution without interpolation and plotted on the Talairach-transformed brain; Talairach coordinates are reported as TAL x, y, z.

## Results

### Behavioral Results

**Figure 1:**
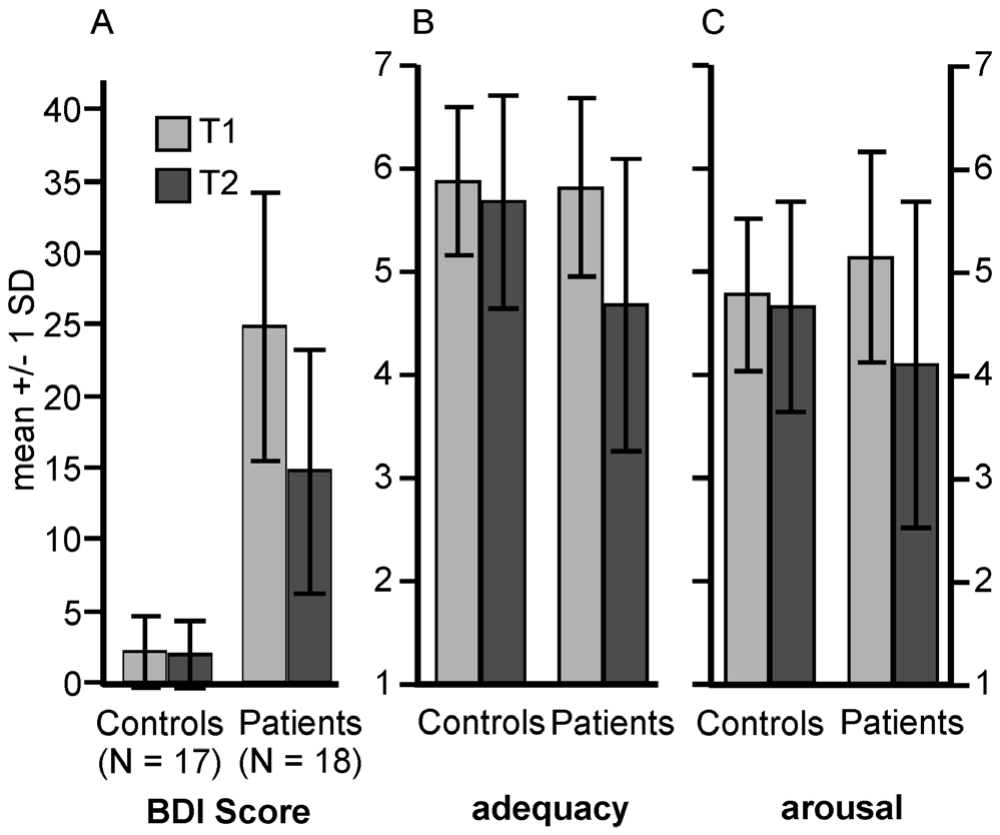
Depression and impact of OPD sentences at T1 and T2. A: BDI (Beck Depression Inventory) scores, B and C: rating of OPD sentences with respect to adequacy (B) and emotional arousal (C). Error bars show +/− 1 SD.

Behavioral data are illustrated in figure 1. At T2 BDI scores had decreased in patients but not in controls (GROUP, F(1,33)  = 85.88, p<.001; interaction GROUP × TIMEPOINT: F(1,33)  = 17.64; p<.001).

At T2 OPD sentences were less adequate and less arousing (according to self-rating) to the patients compared to T1, whereas assessments did not change in controls (adequacy: GROUP × TIMEPOINT interaction, F(1,33)  = 8.39; p<.007; arousal: interaction, F(1,33)  = 8.01; p<.008).

BDI scores in the patient group were not correlated to either the OPD adequacy or arousal scores. The correlation between OPD adequacy and arousal was weak and not significant (r = .39, p<.15).

### fMRI Results

**Figure 2:**
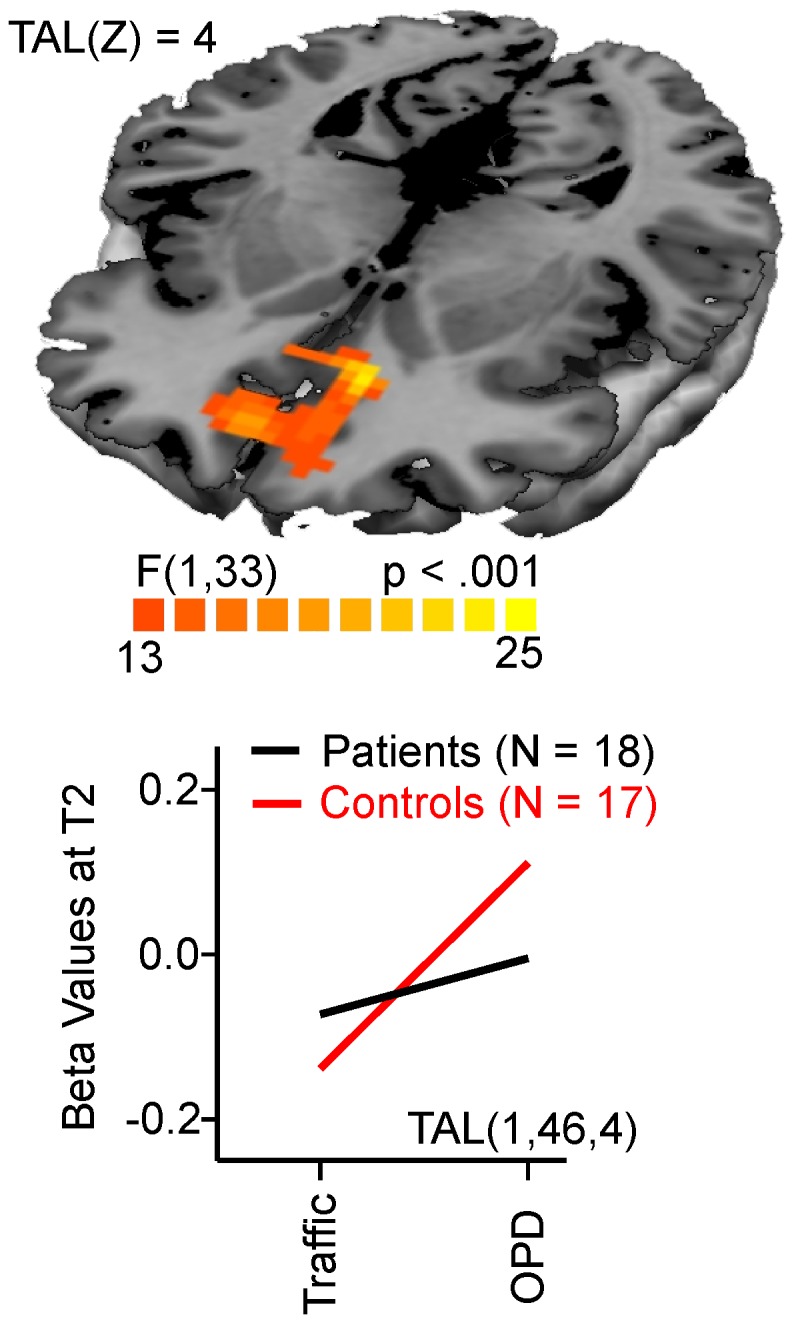
Interaction effect CONDITION × GROUP at T2. Interaction plots are given for the active cluster based on beta values for OPD and traffic sentences, p<.001, cluster threshold 16 voxels.

Main effects of condition with stronger activations for OPD relative to traffic sentences were found in a large occipital cluster (center of gravity TAL −2, −71, 4), the anterior cingulate cortex (TAL −2, 42, 2), and superior frontal gyrus (TAL 18,35,45). There was also activation in two bilateral cluster comprising the putamen and the lateral globus pallidus and extending to the thalamus (TAL 23, −22, 4 and −25, −20, 1, respectively), and in the superior/medial frontal gyrus (TAL −11,48,39). This pattern was similar to that found at T1 [13]. As shown in figure 2, at T2, a group by condition interaction was found for a part of the medial frontal gyrus/Anterior Cingulate (TAL 1,46,4; BA 32,10,24). Although both groups showed increased activation to OPD relative to traffic sentences (see main effect of CONDITION), the difference between OPD and traffic sentences was less pronounced in patients at T2.

**Figure 3:**
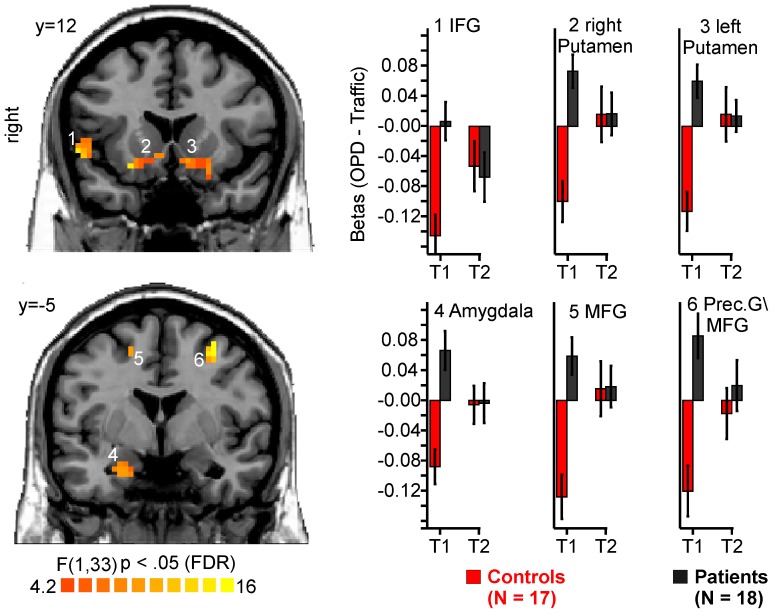
ROI-Analysis; GROUP × CONDITION × TIMEPOINT interaction. Clusters were defined by GROUP × CONDTION interaction at T1. The diagram depicts beta values at T1 and T2. p<.05 FDR corrected, Cluster-threshold 16 voxels.

In the T1-restricted ROI analysis which examined regions that had shown a group by condition interaction at T1, a significant group by condition by time-point interaction was found in several regions as illustrated in figure 3. In all clusters stronger activations to OPD sentences in patients at T1 were no longer seen at T2.

## Discussion

Prior to psychotherapy, patients with depression of the current study had shown hyperactivity relative to healthy controls in limbic and subcortical regions (e.g. amygdala, basal ganglia) when they were confronted with their individual dysfunctional relations associated with their depression [13]. In the follow-up reported here this relative hyperactivity disappeared after eight months of psychodynamic therapy with a focus on current dysfunctional relationship patterns. The patients' brain activity before therapy is in line with a wealth of studies [1], [2], [3], supporting the validity of our novel experimental approach with individualized and clinically-derived OPD stimuli. The fact that the OPD sentences were derived from a structured clinical interview, rated by independent experts and approved by the patients as individual “sore spots” (self-rating of adequacy) backs their external, i.e. ecological, validity. The patients' brain activity after therapy is consistent with the results of a recent study on patients with recurrent major depression treated with psychodynamic therapy, which found decrease of limbic activity after 15 months of treatment during the presentation of personalized sentences concerning attachment related themes [21]. The current study demonstrated that changes in limbic reactivity in depression can already be traced after 8 months of psychodynamic psychotherapy if related to current dysfunctional relationship themes that have been shown to maintain depression. Patients' brain activation changes over time found in our study can be understood following a classical approach to “pathological” brain processes in depression [3], [5], [6]: Limbic hyperactivity is the neurobiological marker indicating dysfunctions in emotion processing systems that are intrinsically linked to depression. If the patients improve clinically (as indicated by significantly lower BDI scores in our sample after eight months of psychotherapy) the dysfunctions and consequently the limbic hyperactivity should no longer be apparent (“normalization”). While reviews of neural changes after psychotherapy have reported inconsistent results [8], [11], our results support the proposed models of neurobiological effects of psychotherapy [3], [5], [6] and confirm studies showing normalization of limbic hyperactivity after psychotherapy [22].

We briefly focus on the functional significance of the limbic regions being differentially active at T1 but not T2 as revealed by our ANOVA interaction analysis. The amygdala has repeatedly been discussed as being hyperactive in depression [23], [24], [25], [26], [27], [28]. We speculate here that enhanced amygdala activity in depressed individuals at T1 reflects their higher emotional involvement in problematic relations. Hyperactivity in patients before psychotherapy in putamen and caudate nucleus is also plausible according to a recent meta-analysis, where the basal ganglia have consistently displayed increased activity in depression after induction of negative affect [24]. This is not surprising, since the basal ganglia have rich interconnections with limbic structures (including the amygdala) and prefrontal areas, and form part of many cortico-subcortical loops engaged in reward and punishment, affect and motivation [29], [30], [31]. The finding that at least two regions show hyperactivity before psychotherapy and are no longer hyperactive after seven months of psychotherapy supports the model of hyperactive limbic regions in depression and “normalization” thereof after psychotherapy [32]. The second main finding, revealed by whole-brain exploratory analysis of existing differences between patients and healthy controls at T2, may lead to an alternative approach to interpret brain activation. At T1, in both groups large areas of the medial prefrontal cortex were active when confronted with OPD sentences. At T2, there was still a major activity in those regions for the same contrast but less so for patients. Considering the involvement of the medial prefrontal cortex in self-referential processing in depression [33], this could signal a decreased self-focus – a cognitive bias associated with depression – in patients after therapy. In fact, recently Cognitive Behavioural Therapy has been shown to reduce medial prefrontal cortex activity associated with self-referential processing when viewing negative stimuli in patients with depression [34] and reduced activity in the medial prefrontal cortex has been demonstrated after 15 months of psychodynamic psychotherapy in patients with depression when dealing with attachment-related topics [21]. Alternatively, we speculate here that activity in medial prefrontal cortex reflects the regulation of emotions initially necessary to cope with the strongly emotional OPD sentences that is less pronounced (and necessary) in patients after eight months of psychotherapy dealing with the content covered by those sentences. As numerous studies have found, the medial prefrontal cortex is crucial for the down-regulation of limbic and subcortical regions when subjects are exposed to strong emotions [35]. This function is vital for patients with depression who are often overwhelmed by such strong negative emotions. In fact, it has been pointed out that brain alterations in depression might not only reflect the pathological process itself but also compensatory mechanisms [5], [21], [36], [37]. One key aspect of psychodynamic therapy in depression is to help the patients gain insight into their dysfunctional relations (as described in the OPD sentences) such that they no longer represent a strong source of distress. Alternatively, patients could have “accepted” their interpersonal problems over the course of psychotherapy to the extent that confrontation with them does not call for such urgent attention and emotion regulation. At T2, patients were in fact significantly less emotionally aroused when confronted with the relationship patterns (self-rating of emotional arousal). For controls, the OPD sentences still provide comparable emotional arousal and lead to brain activity in medial prefrontal cortex, since they received no intervention helping them to cope with their stressful interpersonal situations. In this interpretation, activity in medial prefrontal cortex at T1 could reflect a compensatory mechanism to cope with strong emotional stimuli that is less needed when patients “worked” with the problematic content in psychotherapy over eight months leading to less self-rated arousal and consequently less need to regulate emotions. The interpretation that brain changes were induced by working through dysfunctional relations expressed in the OPD stimuli over the course of psychotherapy points to a limitation of the study design. Although study therapists met on a regular basis to foster adherence and aimed at considering their patients' dysfunctional relations, this could not provide full control over what exactly happened within each therapy session. The lack of an exact and standardized treatment protocol is common for psychodynamic therapy (as opposed to some forms of cognitive behavior therapy) and could not be changed without sacrificing individual therapists' degrees of freedom. Since this is among the first studies investigating neural correlates of psychodynamic therapy in depression, the focus is primarily on effectiveness in a “naturalistic” setting (as opposed to the strict criteria applied in randomized controlled trials). Future investigations building on this study should consider the application of manualized forms of psychodynamic therapy to increase coherence.

Considering the main limitation of our study, the major result – normalization of pre-treatment hyperactivity – could as well be explained by regression-to-the-mean effects. This is a known issue in the literature [38] and has also influenced the discussion of results similar to ours in the study by Fu and colleagues [22]. This possible confound is hard to tackle with statistical means, though, and could only be refuted with a control group of untreated patients with depression measured twice. Ethical considerations ruled out this change in study design for obvious reasons. With this limitation in mind, we cannot infer that patients' changes in brain activity measured over eight months are in fact a causal result of the psychotherapeutic intervention or due to a natural course of depression. Considering the relatively high placebo response rates in major depression [39], spontaneous remission from depressive symptoms is discussed as an important factor in the long-term view. Normalization of brain activity could therefore as well be an unspecific effect not related to treatment. Our patients have had a relatively long history of recurrent depressive episodes and unsuccessful treatments before inclusion in our study. Since for patients with comparable severity of depression remission rates under treatment are substantially lower [40], it is less likely that our study patients showed spontaneous remissions within the eight-month observation period. Arguing in favor of specific effects of our study intervention, the efficacy and effectiveness of psychodynamic therapy, which can best be compared to our approach and its superiority over waiting-list controls has been reported in two meta-analyses [41], [42] (But see [43] for a critical discussion). Additionally, fMRI measurement times were scattered throughout the year to minimize seasonal effects in the course of depression. Finally, the differential pattern found for the interaction effect of condition × group at T1 (both groups with high MPFC activity) and T2 (less increase in MPFC activity for patients) cannot be due to a regression to the mean effect, which would affect both groups alike.

In summary, our results confirm the model of limbic hyperactivity in depression that normalizes after treatment in the case of psychodynamic therapy with novel stimuli that are individually tailored and reflect clinically relevant content. Moreover, less activity in presumably compensatory networks (medial prefrontal cortex) for patients after working with the relevant issues in psychotherapy points to exciting new ways of designing future studies with individualized stimuli.
